# The subcortical default mode network and Alzheimer’s disease: a systematic review and meta-analysis

**DOI:** 10.1093/braincomms/fcae128

**Published:** 2024-04-10

**Authors:** Sara Seoane, Martijn van den Heuvel, Ángel Acebes, Niels Janssen

**Affiliations:** Department of Complex Traits Genetics, Center for Neurogenomics and Cognitive Research (CNCR), Amsterdam Neuroscience, Vrije Universiteit Amsterdam, Amsterdam 1081 HV, The Netherlands; Institute of Biomedical Technologies (ITB), University of La Laguna, Tenerife 38200, Spain; Instituto Universitario de Neurociencia (IUNE), University of La Laguna, Tenerife 38200, Spain; Department of Complex Traits Genetics, Center for Neurogenomics and Cognitive Research (CNCR), Amsterdam Neuroscience, Vrije Universiteit Amsterdam, Amsterdam 1081 HV, The Netherlands; Department of Child and Adolescent Psychiatry and Psychology, Section Complex Trait Genetics, Amsterdam Neuroscience, Vrije Universiteit Medical Center, Amsterdam UMC, Amsterdam 1081 HV, The Netherlands; Institute of Biomedical Technologies (ITB), University of La Laguna, Tenerife 38200, Spain; Department of Basic Medical Sciences, University of La Laguna, Tenerife 38200, Spain; Institute of Biomedical Technologies (ITB), University of La Laguna, Tenerife 38200, Spain; Instituto Universitario de Neurociencia (IUNE), University of La Laguna, Tenerife 38200, Spain; Department of Cognitive, Social and Organizational Psychology, University of La Laguna, Tenerife 38200, Spain

**Keywords:** default mode network, subcortical, functional connectivity, Alzheimer’s disease

## Abstract

The default mode network is a central cortical brain network suggested to play a major role in several disorders and to be particularly vulnerable to the neuropathological hallmarks of Alzheimer’s disease. Subcortical involvement in the default mode network and its alteration in Alzheimer’s disease remains largely unknown. We performed a systematic review, meta-analysis and empirical validation of the subcortical default mode network in healthy adults, combined with a systematic review, meta-analysis and network analysis of the involvement of subcortical default mode areas in Alzheimer’s disease. Our results show that, besides the well-known cortical default mode network brain regions, the default mode network consistently includes subcortical regions, namely the thalamus, lobule and vermis IX and right Crus I/II of the cerebellum and the amygdala. Network analysis also suggests the involvement of the caudate nucleus. In Alzheimer’s disease, we observed a left-lateralized cluster of decrease in functional connectivity which covered the medial temporal lobe and amygdala and showed overlap with the default mode network in a portion covering parts of the left anterior hippocampus and left amygdala. We also found an increase in functional connectivity in the right anterior insula. These results confirm the consistency of subcortical contributions to the default mode network in healthy adults and highlight the relevance of the subcortical default mode network alteration in Alzheimer’s disease.

## Teaser

Seoane *et al*. find the most consistently identified subcortical default mode network in healthy adults and its disruption in Alzheimer’s disease. Through systematic review, meta-analysis and network analysis, they identify a left-lateralized functional connectivity decrease, particularly in the medial temporal lobe and amygdala, in Alzheimer’s disease.

## Graphical Abstract

**Graphical Abstract:**
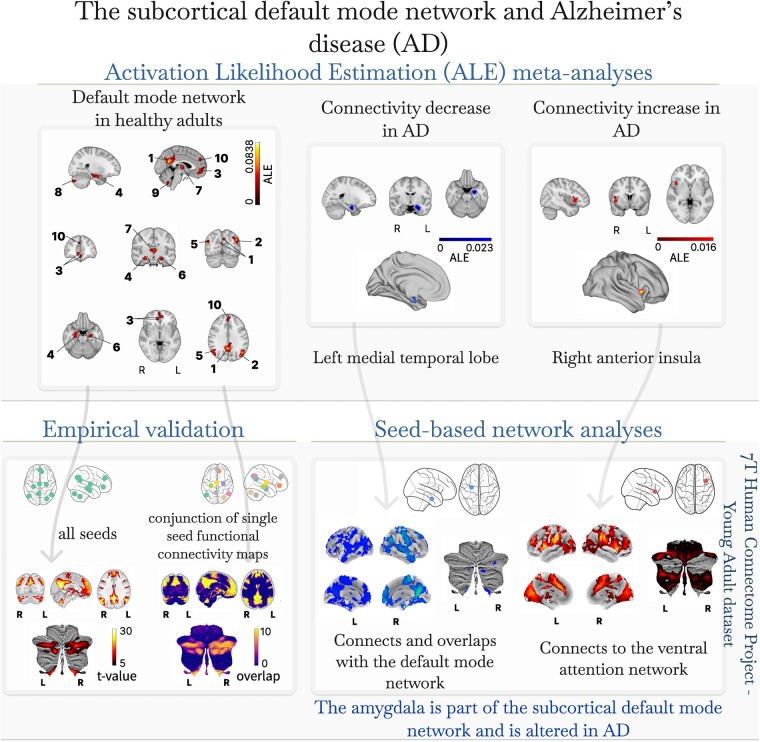

## Introduction

Our understanding of the default mode network emerged from the observation of a characteristic brain spatial pattern of increased blood flow during resting wakefulness and decreased blood flow during externally oriented tasks.^1,2^ The default mode network is considered the most prevalent brain pattern of coactivation during resting state^3-7^; a network crucially implicated in mental and neurological disorders such as schizophrenia,^8^ multiple sclerosis^9^ and Alzheimer’s disease^10,11^; and a suggested target for interventions and biomarkers in Alzheimer’s disease.^12,13^ Our understanding of its cortical components has greatly advanced.^14-18^ However, the study of the subcortical components of the network and their alterations, particularly in the context of Alzheimer’s disease, has not yet derived a clear list of subcortical structures. This study investigates the subcortical regions within the default mode network and their functional connectivity alterations in Alzheimer’s disease.

Previous studies identified subcortical brain regions within the default mode network.^19-22^ Among the early studies into resting-state brain blood flow spatial patterns, the amygdala and the cerebellum were identified as components of what is now recognized as the default mode network.^2,23^ An increasing number of recent studies find subcortical regions with functional and anatomical connections to the default mode network, including the amygdala, anterior and mediodorsal thalamus, basal forebrain, nucleus accumbens, medial septal nucleus, ventral tegmental area, dorsal raphe nucleus, dopaminergic nuclei of the brainstem, caudate nucleus, hypothalamus and cerebellum.^3,21,24-31^ Several of these subcortical structures, such as the basal forebrain, the brainstem, amygdala and thalamus, are not just part of the network but actively modulate the activity of the default mode network and its interplay with other large-scale networks.^26,27,32^

Given the known impact of Alzheimer’s disease on subcortical structures^33-37^ and the default mode network,^11,37,38^ it is crucial to identify the subcortical regions of the default mode network that are altered in Alzheimer’s disease. The earliest signs of Alzheimer’s disease pathology manifest as tau aggregations in the locus coeruleus, even in individuals as young as 10–20 years old.^33^ This alteration progressively impacts other subcortical regions, the medial temporal lobe and, eventually, cortical regions linked to the default mode network.^33,39^ The spreading of amyloid-beta aggregation concurrently progresses from cortical default mode network regions^40^ to regions of the medial temporal lobe and subcortical regions.^33,39,41^ Despite the unclear progression sequence, early stages of the disease significantly affect various subcortical brain regions, including the brainstem, cerebellum, limbic and anterior thalamus, caudate nucleus, putamen and amygdala.^34,42-46^

Functional connectivity of the default mode network and subcortical regions is altered in Alzheimer’s disease and tightly linked to protein pathology.^39,40,47^ Previous studies have predominantly observed decreased functional connectivity in the default mode network^47,48^ and variable changes in its subnetworks.^49,50^ The hippocampus displays both increased and decreased functional connectivity with brain regions including the default mode network.^51-53^ The amygdala, the thalamus and Crus II and lobule IX of the cerebellum show reduced functional connectivity with default mode network regions.^35,36,54^ Notably, these regions also exhibit hallmark Alzheimer’s disease pathology.^40,44,45,47,55^ A recently proposed model describes the intertwined relationship between these alterations in two regions of the default mode network: the medial temporal lobe and the posteromedial cortex.^39^ This interplay leads to a cycle of atrophy, hyperexcitability and further tau pathology accumulation, particularly impacting regions within the medial temporal lobe, an area particularly vulnerable in Alzheimer’s disease.^39,44,56^ Therefore, characterizing the functional connectivity alterations within the network could be crucial for identifying treatment targets.

Regardless of numerous advances in the field, our understanding of the subcortical components of the default mode network, as well as of the subcortical default mode network alterations in Alzheimer’s disease, remains incomplete. The lack of consistent results may be linked to at least three main reasons. First, a branch of neuroimaging analysis methods based on the projection of the cortical data from a volume to a surface has improved our cortical spatial precision.^57^ However, this often results in the neglect of subcortical regions and the hippocampus, unless used in combination with volume-based methods.^58^ Second, the signal-to-noise ratio in magnetic resonance images is notably lower in some areas of the brain, including subcortical structures.^59,60^ Given that the detection of functional connectivity between brain regions is influenced by the quality of the signals,^24^ the regional difference in signal quality could impact our ability to detect functional connectivity. Third, subcortical structures are small and variable, which makes it difficult to align them for group analysis.^20^ Thus, conducting a systematic study of the findings of subcortical default mode network components and their alterations in Alzheimer’s disease is a crucial task not yet done, key to identifying the most consistent regions across studies.

In this study, we performed a systematic review, meta-analysis and empirical validation of cortical and subcortical default mode network regions in healthy individuals. We also conducted a systematic review, meta-analysis, seed-based network analysis and conjunction analysis of the cortical and subcortical brain sites that present functional connectivity alterations in Alzheimer’s disease and their connectivity with default mode network regions (see Table 1). We reduced bias in examining the subcortical default mode network alterations in Alzheimer’s disease by meta-analysing the brain sites that show altered functional connectivity to any brain region in the disease and then determining whether these brain sites were part of the default mode network in healthy normal conditions. We expected to find subcortical structures of the default mode network including the thalamus, caudate nucleus, amygdala, Crus I/II and lobule IX of the cerebellum, brainstem and basal forebrain. Another anticipation was to identify brain cortical and subcortical sites of decreased functional connectivity in Alzheimer’s disease that included the precuneus, hippocampus, thalamus, brainstem and cerebellum. Identifying the consistent involvement of subcortical structures in the default mode network, the changes in functional connectivity in Alzheimer’s disease as well as the link between the brain sites of alteration and networks has the potential to impact our knowledge of the network’s role in the disease.

**Table 1:** Summary of Population, Intervention, Comparator, and Outcome (PICO) components per question

	Default mode network in healthy participants	FC alterations and their directions in Alzheimer’s disease
Population	Healthy participants	Patients that meet the criteria for possible or probable Alzheimer’s disease
Intervention	FC analysis of the default mode network or its seeds performed on whole-brain data	Comparison of whole-brain FC between Alzheimer’s disease and matched HC groups (Alzheimer’s disease > HC and Alzheimer’s disease < HC)
Comparator	None needed	Healthy elderly controls
Outcome	What are the cortical and subcortical nodes of the default mode network?	What are the brain sites of increases and decreases in FC in Alzheimer’s disease? How do they relate to the default mode network?

HC, healthy controls; FC, functional connectivity.

## Materials and methods

### Search strategy and database selection

**Figure 1:**
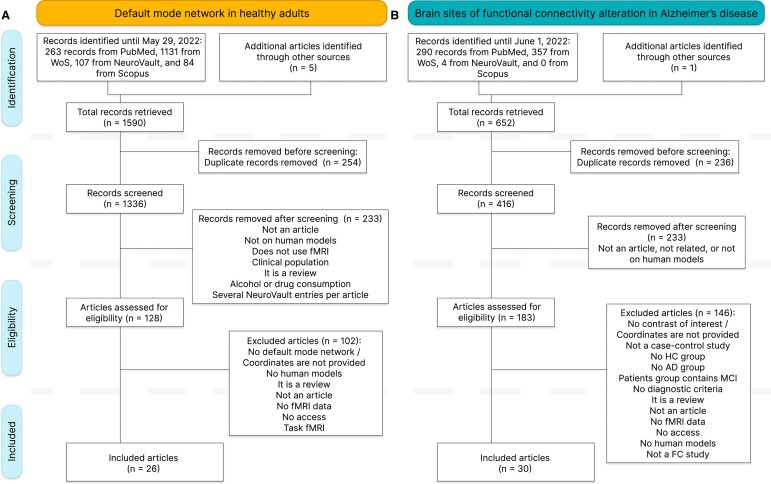
**PRISMA workflows.** Workflows for (**A**) the default mode network in healthy adults and for (**B**) functional connectivity alterations in Alzheimer's disease. The workflow is divided into four phases (from top to bottom): identification, screening, eligibility and inclusion. In the identification phase, the initial search retrieved 1586 records for question 1 and 652 records for question 2. These records were reduced in the screening and eligibility phases to 26 and 30 articles that were used in this meta-analysis.

This study follows the Preferred Reporting Items for Systematic Reviews and Meta-Analyses (PRISMA) guidelines^61^ and uses PubMed, Scopus, Web of Science (WoS) and NeuroVault^62^ for database searches. The search was conducted between 4 May 2022 and 1 June 2022 and included the ‘All Fields’ option in PubMed; title, abstract and keywords in Scopus; and title, abstract, keywords and automatically generated terms from the titles of the cited papers in WoS. We did not limit our search to a specific timeframe. The search details, including queries, search dates and number of results are documented in Supplementary Tables 1 and 2. We also scrutinized the reference sections and the studies previously known to the authors for complete coverage (see Fig. 1).

### Selection criteria

Study search and selection were performed by S.S. and revised by N.J. For the healthy adult default mode network meta-analysis, whole-brain studies that examined the intrinsic functional connectivity of the default mode network in healthy adults (18–55 years old) and which reported results in standard anatomical space (Montreal Neurological Institute, MNI, or Talairach) were included. To include as many articles for the meta-analysis as possible while excluding articles with extremely small sample sizes, we selected studies with at least 15 participants. Only studies that examined whole-brain functional MRI (fMRI) data (using volume-based or combined surface- and volume-based methods) were considered. This excluded, for example, studies that only included cortical regions in their analysis. Coordinates reported in the manuscripts as part of the default mode network or connected to its most central brain regions (i.e. the precuneus/posterior cingulate cortex/retrosplenial cortex) were included in the analysis. Negative functional connectivity or ‘anticorrelations’ were not considered for the analysis. Resting-state fMRI studies were considered for inclusion, regardless of whether they had closed or open eyes and whether there was a visual fixation.

For the Alzheimer’s disease coactivation alteration meta-analysis, included studies were case-control studies that examined whole-brain functional connectivity differences between Alzheimer’s disease (AD) patients and healthy controls (HC). Only records in which the patients met the criteria for possible or probable Alzheimer’s disease published by the National Institute of Neurological and Communicative Diseases and Stroke/Alzheimer’s Disease and Related Disorders Association (NINCDS-ADRDA) or the National Institute on Aging—Alzheimer’s Association (NIA-AA) were included. All included studies had at least 10 participants in both patients and control groups. We used a lower sample size threshold taking into account that samples in case-control studies tend to be low. All experiments from the selected articles that consisted of Alzheimer’s disease > HC or Alzheimer’s disease < HC contrasts were included. Finally, the quality of the studies included in the meta-analysis of Alzheimer’s disease brain sites was assessed using the Critical Appraisal Skills Programme (CASP^63^) checklist for case-control studies.

### Data extraction

For the healthy adult default mode network meta-analysis, the extracted data included sample size, age, gender, network extraction technique and coordinate space in which the coordinates are provided in the study, presence of subcortical regions in the results and coordinates of the default mode network (see Supplementary Table 3). Variables extracted from the included studies in the Alzheimer’s disease coactivation alteration meta-analysis were sample size, gender, age, diagnostic criteria for Alzheimer’s disease, Clinical Dementia Rating (CDR) scores, Mini-Mental State Examination (MMSE) scores, Montreal Cognitive Assessment (MoCA) scores, years of education, biomarkers used, apolipoprotein E (APOE) alleles, main analysis technique used and coordinates for brain sites with altered functional connectivity in Alzheimer’s disease compared with HC (see Supplementary Tables 4 and 5 and Supplementary Fig. 1).

The extracted data included sets of coordinates, or foci, in either MNI or Talairach space. These foci were extracted from the manuscripts, Supplementary Methods and openly available volumes. Each included article could have multiple functional connectivity maps for the default mode network or sets of functional connectivity alteration sites in Alzheimer’s disease. Each functional connectivity or contrast map was treated as an independent experiment in the meta-analysis. See more in the Supplementary Methods section.

### Statistical analysis

#### Meta-analysis procedure

Three meta-analyses of brain maps were performed: first, a meta-analysis using foci from experiments of the default mode network in healthy adults; second, a meta-analysis using foci with increased functional connectivity in Alzheimer’s disease patients against HC (Alzheimer’s disease > HC); and third, a meta-analysis of foci with decreased functional connectivity in Alzheimer’s disease patients against HC (Alzheimer’s disease < HC). Separate foci files were prepared for the three meta-analyses, and activation likelihood estimation (ALE) was calculated using the GingerALE software.^64,65^ ALE meta-analysis is a coordinate- and kernel-based method for meta-analysis of neuroimaging data. This method consists of three main steps: first, the interpolation of a three-dimensional Gaussian distribution (or kernel) around each focus (set of three coordinates): second, a combination of the resulting maps for each experiment; and third, the calculation, through a non-additive random effects model, of a single combined map that represents the consistent activations across experiments. This model uses the sample size per experiment, given its impact on statistical power, to find statistical effects. Moreover, the significance of this map is assessed by a Monte Carlo test under the null hypothesis of complete spatial randomness.^66-68^ The three meta-analyses were performed using the default cluster-level family-wise estimation (FWE) correction at *P* < 0.01, 1000 permutations and thresholding of *P* < 0.001.

#### Data set used for empirical validation and network analysis

A resting-state fMRI sample was used in both the validation analysis of the default mode network brain regions and the network analysis from the altered brain sites in Alzheimer’s disease. This data set consisted of resting-state fMRI data from 172 healthy young participants in the 7T WU-Minn Human Connectome Project’s 1200 subjects’ data set. These participants were between 22 and 35 years old, 60% female. Please find more information in the Supplementary Methods and elsewhere.^69^ The data analyses were conducted in agreement with the declaration of Helsinki and with the protocol established by the Ethics Commission for Research of the Universidad de La Laguna, the Comité de Ética de la Investigación y Bienestar Animal.

#### Empirical validation of the cortical and subcortical default mode network

The brain regions identified in the healthy adult default mode network meta-analysis served as seeds to calculate new functional connectivity maps. Spherical regions of interest (ROIs) were created around the peak ALE MNI coordinates of the brain regions resulting from the meta-analysis of the default mode network in healthy adults using FSL’s (FMRIB Software Library) fslmaths. Spheres had a radius of 4 mm each, and the average time series of the voxels inside each sphere was extracted for each subject and resting-state session using FSL’s fslmeants. Each average time series was regressed to the individual whole-brain resting-state fMRI data in a general linear model with fsl_glm program. Group-level functional connectivity analysis was performed for each ROI using FSL’s randomise and consisted of randomized non-parametric voxel-wise one-sample *t*-tests (5000 permutations), threshold-free cluster enhancement and *P* < 0.001 as the statistical threshold. A conjunction analysis of the group functional connectivity maps was performed using the Analysis of Functional NeuroImages’s (AFNI) 3dcalc function to determine the overlap between the functional connectivity maps of individual ROIs. An additional seed-based functional connectivity analysis was performed using the same procedure as with each individual ROI but regressing the average time series across the 10 seeds. The group-level map was also obtained using randomise with randomized non-parametric voxel-wise one-sample *t*-tests (5000 permutations), threshold-free cluster enhancement and *P* < 0.001 as the statistical threshold.

### Network analysis of functional connectivity alterations in Alzheimer’s disease

The connection between the meta-analytical brain sites of functional connectivity alteration in Alzheimer’s disease and the default mode was assessed through a seed-based analysis following the same procedure as per the empirical validation of the default mode network brain regions, except that this time no conjunction analysis was performed across functional connectivity maps of alteration sites. We performed this analysis on the same sample of healthy young adults to identify the large-scale networks to which the altered brain sites belong under normal healthy conditions.

## Results

### Filtered search results

Systematic searches were performed separately for studies related to the default mode network in healthy adults and to the functional connectivity case-control studies in Alzheimer’s disease. The 2 independent searches retrieved 1585 and 651 records, respectively. Six additional articles were identified and included in the lists, for a total of 1590, and 652 records. After the removal of duplicate records, a total of 1336 records remained from the default mode network in healthy adults searches and a total of 417 from the case-control studies searches. The first screening was performed to filter records that were not related to the questions, were not human model studies, did not use fMRI, studied clinical populations or were not scientific research articles. A total of 128 articles of the default mode network in healthy adults and 183 articles of the Alzheimer’s disease functional connectivity differences were assessed for eligibility, and 26 and 30 studies were included in the meta-analyses (see Fig. 1).

For the healthy adult default mode network meta-analysis, a total of 852 foci from 55 experiments in 26 articles were used. The sample sizes ranged from 15 to 500 (median = 59), with weighted pooled proportion (pP) of 49% males and weighted pooled mean (pM) age of 29.28 years old (20.63–42.30 years old). The total sample size across all these experiments was 5165. See Supplementary Table 3 for a more detailed description of the sample.

The meta-analysis of the Alzheimer’s disease < HC contrast included 40 experiments from 26 studies. For each experiment, we used the smallest sample size between case and control groups to weight the neuroimaging meta-analysis, leading to a total sample size of 927. The number of participants with Alzheimer’s disease per experiment ranged from 10 to 70 (median = 20.71; pP = 45% male), and the number of HC ranged from 10 to 174 (median = 32.88; pP = 44% male). Alzheimer’s disease participants were 71.03 years old, and HC were 68.34 years old, on average. CDR scores were reported in 17 studies, with a CDR score 0 in HC and 0.9 (range of 0.5–1.3) in Alzheimer’s disease. MMSE scores were reported in 20 studies, with a pM 28.82 for the HC group and 20.43 for the Alzheimer’s disease group. HC had 12.43 years of education and Alzheimer’s disease had 10.69. Data on additional cognitive tests (e.g. MoCA, AVLT) and biomarkers were reported in some studies, but the reports were either too variable or scarce to make a global summary. The predominant diagnosis among the included studies was probable Alzheimer’s disease (17 studies, 12 being exclusively probable Alzheimer’s disease), alongside reports of possible Alzheimer’s disease, mild Alzheimer’s disease and Alzheimer’s disease. Six studies, based on McKhann *et al*.^70^ criteria or unspecified NINCDS-ADRDA guidelines, might have included some patients with mild cognitive impairment, a point discussed in McKhann *et al*.^71^ For detailed information per study, please see Supplementary Table 4 and Supplementary Fig. 1.

The meta-analysis of the Alzheimer’s disease > HC contrast included 20 experiments from 15 studies. Sample sizes in the Alzheimer’s disease group ranged from 10 to 70 (median = 19), with an average age of 73.69 years old, and 46% being male. Sample sizes in the HC groups ranged from 10 to 67 (median = 16.50), with an average age of 70.68 years old, 48% male. Considering the smallest group size for each case-control pair, the total sample size for the neuroimaging meta-analysis was 426. CDR scores were reported in 10 studies and were 0 for HC and 0.83 for Alzheimer’s disease (range of 0.5–1.26). Thirteen studies reported MMSE scores, with a pM 28.66 for HC and 21.79 for Alzheimer’s disease. Eleven studies provided data on the years of education, with 13.16 years in HC and 21.79 in Alzheimer’s disease. Seven of the 15 studies reported probable Alzheimer’s disease diagnosis, with one of them indicating possible or probable Alzheimer’s disease diagnosis. See Supplementary Table 5 and Supplementary Fig. 1 for detailed information per study. The included studies did not report any specific subtypes of Alzheimer’s disease.

### Cortical and subcortical components of the default mode network

**Figure 2:**
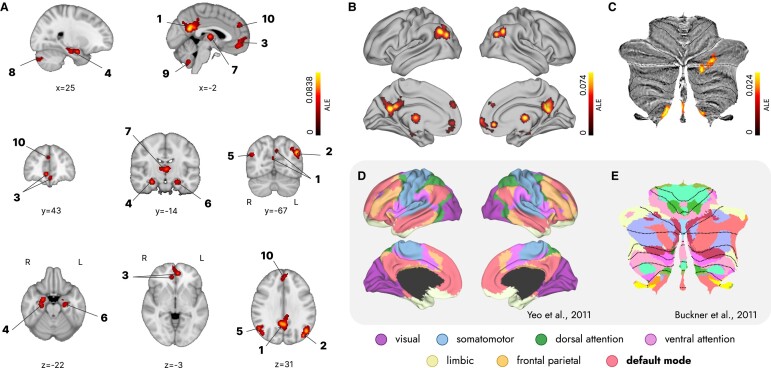
**Cortical and subcortical brain regions of the default mode network.** (**A**) The left panel displays the 10 spotted cortical and subcortical clusters of the resting-state default mode network on the standard MNI152 volume, determined by ALE meta-analysis with a cluster-level FWE correction of *P* < 0.01 (1000 permutations) and a cluster-forming threshold of *P* < 0.001. The same clusters are projected to a cortical surface (**B**) and to a cerebellar flatmap (**C**) for comparison with the default mode network represented on panels **D** and **E**. In panel **D**, the Yeo *et al*.^14^ 7 resting-state network parcellation is displayed on a cortical surface, and in panel **E**, the cerebellar network parcellation by Buckner *et al*.^86^ is displayed on a cerebellar flatmap.^72^

The healthy adult default mode network ALE meta-analysis resulted in 10 consistent clusters. These clusters covered the (extensively documented) cortical posterior and anterior cingulate cortices, precuneus, inferior parietal lobule, angular gyrus, medial frontal pole, ventromedial prefrontal cortex, anterior and middle parahippocampal gyrus and hippocampus. Regarding the main focus of this study, the clusters also covered the subcortical thalamus, amygdala and Crus I/II and lobule and vermis IX of the cerebellum (see Table 2 and Fig. 2). Contributions from cortical regions, thalamus and cerebellar lobule IX to the default mode network were bilateral, and cerebellar Crus I/II contributions were right-lateralized.

**Table 2:** Clusters coverage in the resting-state default mode network in healthy adults

Cluster number	Brain regions covered	Lateralization	Coordinates in MNI152 space (peak ALE value)	ALE	*P*-value	*z*-value
1	Posterior cingulate gyrus, precuneus	Bilateral (65.2% L; 34.8% R)	−4 −56 22	0.083	2.2920986E^−19^	8.92
2	Inferior parietal lobule, angular gyrus	Left (100% L)	−48 −66 34	0.081	9.648886E^−19^	8.76
3	Medial frontal pole	Bilateral (55.5% L; 44.5% R)	8 42 −6	0.050	3.8794934E^−10^	6.15
4	Parahippocampal gyrus, hippocampus, amygdala	Right (100% R)	28 −14 −20	0.050	2.5647295E^−10^	6.22
5	Inferior parietal lobule, angular gyrus	Right (100% R)	52 −60 34	0.062	2.4539084E^−13^	7.23
6	Parahippocampal gyrus, hippocampus, amygdala	Left (100% L)	−28 −24 −12	0.048	1.2546583E^−9^	5.96
7	Thalamus	Bilateral (62.8% L; 37.2% R)	−2 −12 6	0.051	2.144407E^−10^	6.24
8	Crus I/II of the cerebellum (pyramis, inferior semilunar lobule and uvula)	Right (100% R)	30 −78 −34	0.037	3.5167733E^−7^	4.96
9	Lobule and vermis IX of the cerebellum	Bilateral (76.7% L; 23.3% R)	−6 −56 −46	0.040	7.92126E^−8^	5.24
10	Ventromedial prefrontal cortex, anterior cingulate cortex	Bilateral (87.9% L; 12.1% R)	0 50 32	0.042	2.4101716E^−8^	5.46

ALE, activation likelihood estimation; dlPFC, dorsal lateral prefrontal cortex; L, left; MNI, Montreal Neurological Institute; R, right; SMA, supplementary motor area.

### Brain sites of functional connectivity alterations in Alzheimer’s disease

**Figure 3:**
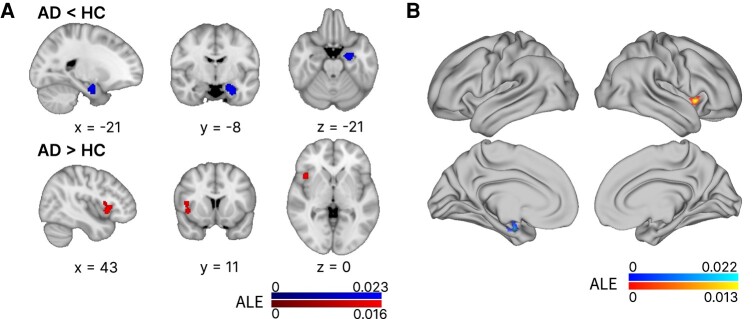
**Brain sites of functional connectivity alterations in Alzheimer’s disease.** (**A**) This figure shows a volume view in standard MNI space of the ALE clusters with increased and decreased functional connectivity in Alzheimer’s disease. The cluster in red indicates increases in Alzheimer’s disease against controls, and the cluster in blue indicates decreases. In **B**, the same clusters are projected to the average surface for comparison with the resting-state networks displayed in Fig. 1A. These clusters were identified through ALE meta-analysis with a cluster-level FWE correction of *P* < 0.05 (1000 permutations) and a cluster-forming threshold of *P* < 0.001.

One cluster of functional connectivity decrease and another cluster of functional connectivity increase were found in Alzheimer’s disease compared with controls (see Fig. 3). The brain site of decreased coactivation was 100% left-lateralized and covered the parahippocampal gyrus, amygdala and hippocampus. It had a maximum ALE value at −26, −8 and −26 MNI coordinates (ALE = 0.023; *P*-value = 8.412384E^−7^; *z*-value = 4.79). The cluster of increased coactivation was 100% right-lateralized and covered the anterior insula and some of the precentral gyrus, with a maximum ALE value of 0.0164 at 42, 14 and −2 MNI coordinates (*P*-value = 3.911245E^−6^; *z*-value = 4.47).

### Empirical validation of the default mode network clusters

**Figure 4:**
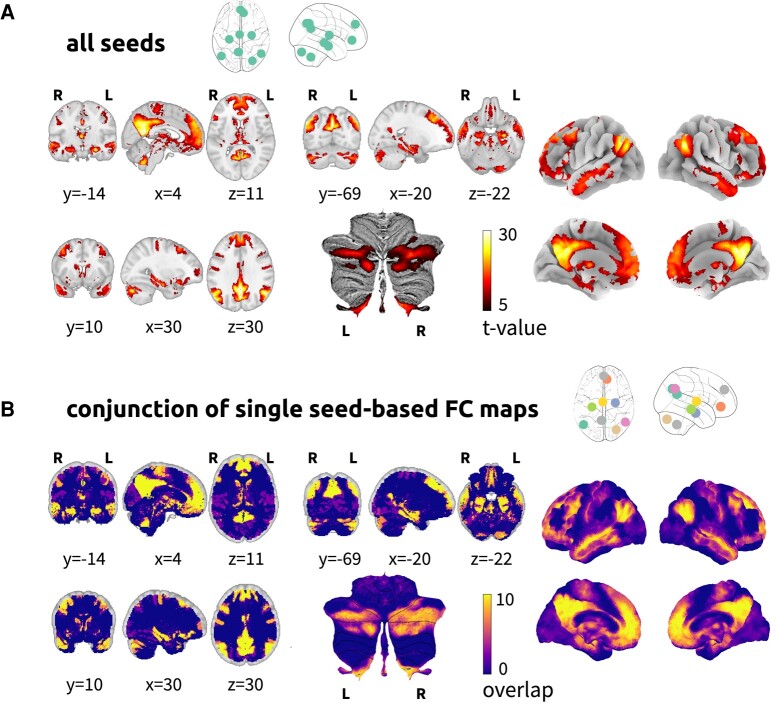
**Validation analysis of the default mode network in the HCP 7T data set.** (**A**) A group functional connectivity map is displayed in panel **A** for the functional connectivity map to the mask containing all clusters (voxel-wise 1-sample *t*-tests with 5000 permutations and threshold-free cluster enhancement at *P* < 0.001). (**B**) Results from the conjunction analysis of the functional connectivity maps from each seeded cluster (see also Supplementary Figs. 5 and 6).

The group map of functional connectivity to all ROIs displayed coactivation with precuneus, posterior cingulate cortex, ventromedial prefrontal cortex, frontal pole, orbitofrontal cortex, anterior insula, inferior parietal cortex, precentral and postcentral gyri, hippocampus, left middle temporal gyrus, left fusiform gyrus, bilateral Crus I/II and in lobule IX of the cerebellum, mediodorsal and pulvinar nuclei of the thalamus, caudate nucleus, amygdala and basal forebrain (see Fig. 4A and Supplementary Table 6). Each meta-analytical seed showed significant functional connectivity to the other default mode network brain regions (see Supplementary Figs. 2 and 3 and Supplementary Tables 7–17). The conjunction analysis of functional connectivity maps of meta-analytical ROIs showed that all seeds connected to the precuneus, posterior cingulate cortex, ventromedial prefrontal cortex, frontal pole, inferior parietal cortex, hippocampus, left middle temporal gyrus and small clusters in the parahippocampal cortex, bilateral Crus I/II and in lobule IX of the cerebellum, anterior insula, basal forebrain and in the pulvinar nuclei of the thalamus (see Fig. 4B; refer to Supplementary Fig. 4 for additional results obtained using less stringent statistical thresholds).

### Networks of the brain sites of functional connectivity alterations in Alzheimer’s disease

**Figure 5:**
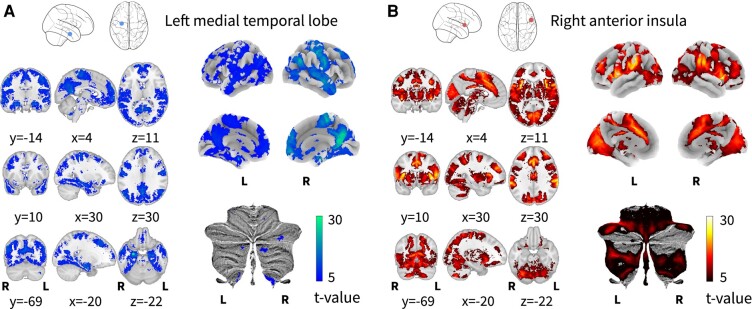
**ROI-to-network analysis of Alzheimer’s disease–altered brain sites.** (**A**) Functional connectivity map from the ALE meta-analysis cluster of decreased functional connectivity. (**B**) Functional connectivity map from the meta-analysis cluster of increased connectivity. Both maps were calculated with a voxel-wise 1-sample *t*-tests with 5000 permutations and threshold-free cluster enhancement at *P* < 0.001.

The seed-based functional connectivity analysis of the decreased functional connectivity site in Alzheimer’s disease showed connectivity to the precuneus cortex, posterior cingulate cortex, ventromedial prefrontal cortex, precentral and postcentral gyri, bilateral hippocampi, amygdala, parahippocampal gyri, superior and middle temporal gyri, temporal pole, inferior parietal cortex, basal forebrain, Crus I/II and lobule IX of the cerebellum and fusiform cortex (see Fig. 5A). The increased functional connectivity site displayed connectivity to the cuneus; intracalcarine and supracalcarine cortex; lingual gyrus; occipital pole; insula; opercular cortex; inferior parietal lobule; intraparietal sulcus; anterior and posterior cingulate cortices; supplementary motor cortex; precentral and postcentral gyri; ventromedial, orbitofrontal and dorsolateral prefrontal cortices; triangularis and opercularis; lobules V, VI, VIIb, VIIIa, IX and Crus II of the cerebellum; putamen; and middle thalamus (see Fig. 5B; see also Supplementary Figs. 5 and 6 for results obtained using less stringent statistical thresholds).

### Conjunction analysis of default mode network and Alzheimer’s disease alterations

Given the meta-analytical clusters for the default mode network and the functional connectivity alteration in Alzheimer’s disease, we observed an overlap between the default mode network’s cluster 6 and the Alzheimer’s disease cluster of decreased functional connectivity. To extract the common cluster of voxels between these two clusters, we thresholded the volumes with ALE values of the default mode network meta-analysis and the meta-analysis of decreased functional connectivity in Alzheimer’s disease at ALE = 0.015 and performed a conjunction analysis, again using the AFNI function 3dcalc. The resulting overlapping cluster consisted of 902 629 voxels that covered the left anterior hippocampus and left amygdala around MNI coordinates −22, −9 and −21.

## Discussion

The default mode network has been extensively studied in the cortex and increasingly studied in the subcortex. However, there still is a lack of consensus on the specific subcortical regions involved in the default mode network and their functional connectivity changes in Alzheimer’s disease. Our study combined systematic review, meta-analysis, empirical validation and network analysis to examine the brain regions conforming the default mode network in healthy adults and those showing altered functional connectivity in Alzheimer’s disease. We identified and validated 10 clusters within the default mode network of healthy adults (5 subcortical clusters). We also found a consistent decrease in functional connectivity in the left anterior hippocampus and left amygdala overlapping with the subcortical default mode network. These findings expand our understanding of the default mode network and its relevance to Alzheimer’s disease.

Among the 10 clusters identified in the default mode network, 7 were located in regions typically associated with the cortical components of the default mode network. These include the precuneus, posterior cingulate cortex, inferior parietal lobule, angular gyrus, ventromedial prefrontal and anterior cingulate cortices, medial frontal pole and medial temporal lobe, including the hippocampus.^73^ These regions primarily constitute the medial temporal lobe and core subsystems of the default mode network, involved in mnemonic scene construction (i.e. in building mental scenes from memory).^16^ Our validation analysis identified functional connectivity between these default mode network regions and other cortical default mode network regions, specifically the middle and superior temporal gyri and the dorsal prefrontal cortex,^73^ potentially covering the entire cortical default mode network.

Our results extend beyond well-known cortical brain regions, revealing the consistent inclusion of subcortical brain regions in the default mode network. The thalamus, amygdala and specific areas of the cerebellum—Crus I/II and lobule and vermis IX—were present, which is consistent with previous results. The thalamus has been identified in multiple previous studies.^20,21,74-80^ Our meta-analysis specifically highlights a middle and medial cluster covering the mediodorsal nucleus, and our validation analysis points to a medial and posterior thalamic cluster covering the pulvinar complex. Previous studies find functional connectivity between the default mode network and the anterior mediodorsal thalamus,^20^ the central lateral nucleus,^21^ the paraventricular thalamus^76,78^ and to an anterior portion of the right and a posterior portion of the left thalamus.^81^ Considering this variability and the resolution of the results from this meta-analysis, the specific contributions within the thalamus remain unclear. Brain areas within the cortical default mode network also coactivate with the amygdala^29,82-84^ (with laterobasal, superficial and centromedial nuclei) and the cerebellum (CrusI/II and lobule and vermis IX, with some reports on lobules VIIIB and X).^30,76,85-87^

We identified five consistent subcortical brain regions of the default mode network, acknowledging that other subcortical regions might contribute but remain undetected. Although most studies show cerebellar contributions to the default mode network from the Crus I/II and lobule IX,^76,86,87^ some studies point to vermis X, lobule VIIIB or the dentate gyrus nucleus.^76,87,88^ Also, some previous studies had found functional connectivity with the basal forebrain, the caudate nucleus, the nucleus accumbens and the ventral tegmental area.^20,21^ Also, despite incorporating numerous coordinates from the brainstem in our meta-analyses, the lack of sufficient overlap and consistency among studies, together with the application of stringent thresholds (see Supplementary Fig. 4), possibly prevented the identification of a meta-analytical cluster in this region.

The default mode network regions presented herein are anatomically connected^89-91^ through the cingulum anterior and posterior tracts, the superior and inferior longitudinal fascicles, the arcuate fasciculus, the uncinate fasciculus, the frontal orbitopolar fasciculus and the corpus callosum.^4,20,30,89,92^ Research using diffusion tensor imaging tractography has identified tracts that connect default mode network regions to the thalamus and basal forebrain.^20^ The thalamus is connected to the cortical default mode network by the anterior thalamic radiations, the basal forebrain by the cingulum and fornix, and the thalamus and basal forebrain are connected to each other by the bundle of Vicq D’Azyr. Other diffusion tensor imaging tractography studies define multiple tracts connecting the cerebellum to all brain lobes, including a dentate-pontine-cerebellar tract.^93^ Viral tracing techniques show connections between Crus I and II and the prefrontal cortex ,^94^ and the amygdala also has anatomical projections to the adjacent hippocampus and prefrontal cortex from its basolateral complex.^95,96^

The anatomical connections between the cortical and subcortical structures of the default mode network suggest its overlap with the Papez circuit, an anatomical network linked to emotion in 1937.^20,97-99^ Key regions like the thalamus, posterior cingulate cortex and hippocampal formation are part of this circuit.^97^ Subsequent research on this circuit made it evolve into a more complex network that is relevant for several neurological conditions, such as Alzheimer’s disease.^12,98^ For instance, deep brain stimulation targeting the Papez circuit has shown benefits in overall cognitive performance in Alzheimer’s disease.^12^ Additional structures and tracts have been incorporated into the Papez circuit based on their anatomical connections, including the amygdala and Crus I and II.^100,101^

Default mode network regions display altered functional connectivity in Alzheimer’s disease,^35,36^ but the connection between the subcortical default mode network and Alzheimer’s disease remains unclear. Our study identified a cluster of reduced functional connectivity in the left hippocampus, amygdala and parahippocampal gyrus, as well as the connectivity and convergence of this cluster with the default mode network. This finding resonates with previous findings of reduced functional connectivity in the default mode network in Alzheimer’s disease,^48^ and particularly a left-sided alteration in its posterior subnetwork,^102^ and with findings of the connectivity between these medial temporal lobe regions and the default mode network^29,82-84,103^ (with exceptions^104^). These findings mirror broader pathology associations with the cortical default mode network^40,105^ and also align with a recently proposed model of Alzheimer’s disease.^39^ This model posits a bidirectional relationship between connectivity disruptions within the default mode network and the accumulation of protein pathologies, such as amyloid-beta and tau, with a crucial role of the medial temporal lobe in the intersection of pathologies.

Zooming into the amygdala, we note its multifaceted involvement in Alzheimer’s disease. The amygdala is vulnerable to Alzheimer’s disease pathologies, starting with neurofibrillary tangle pathology as early as in Braak and Braak phase II.^44,106^ Alterations of the amygdala also extend to functional connectivity,^35,104,107^ including a reduction in connectivity between left amygdala and default mode network regions.^108^ This might be especially relevant, taking into account the role of the amygdala as one of various subcortical regions with a causal inhibitory role in the activity of the default mode network.^109^ The left amygdala, as well as the hippocampus, presents volume reductions in Alzheimer’s disease,^110^ which are associated with the carriage of APOE-ε4 alleles.^111^ Furthermore, there is an inverse relationship between tau brain accumulation and left amygdala shape.^112^

The relevance of the amygdala extends to its potential role in the spread of pathology within the medial temporal lobe. Traditional pathways, like the spread of neurofibrillary tangles from the entorhinal cortex to the posterior hippocampus, fail to account for early tangle presence in the anterior hippocampus.^113^ The extensive anatomical and functional connections between the amygdala and anterior hippocampus and other early pathology sites, such as the locus coeruleus, transentorhinal cortex, the subiculum and Cornu Ammonis 1 (CA1), position the amygdala as a hypothesized alternative route for tangle propagation.^113-116^ Our results, alongside referenced studies, suggest that reduced connectivity in the left amygdala and left hippocampus might constitute a neurobiological feature of Alzheimer’s disease and could be a potential biomarker of the disease.^13^ Future studies should explore how the connectivity between the hippocampal/amygdalar cluster and the default mode network correlates with protein aggregation in these areas.

We also identified increased functional connectivity in the right anterior insula, as well as the connectivity of this cluster to a mixture of visual, ventral attention or salience networks’ areas in an ultrahigh magnetic field sample of healthy young adults. Among the often overlapping salience and ventral attention networks, the former is bilateral, while the latter is right-lateralized.^117,118^ These networks show increased functional connectivity in Alzheimer’s disease,^119-124^ associated with preserved or improved social cognition, as well as with irritability and signs of anxiety.^124,125^ The anterior insula is also involved in a system for conscious access to sensory information,^126^ and volume loss and fluorodeoxyglucose (FDG)-PET hypometabolism of the right anterior insula is associated with hallucinations in Alzheimer’s disease.^127^ Furthermore, the anterior insula, along with the locus coeruleus, mediodorsal thalamus and basal forebrain,^27,32,126^ is crucial for transitioning between the default mode network and networks related to external attention and executive control.^126^

We found consistent alteration of brain regions within the default mode and ventral attention networks. These networks compose a proposed brain system for allostasis (i.e. the process of regulating physiological functions to maintain homeostasis), with the amygdala and insula partially overlapping in both component networks.^128-130^ In a speculative context, the overlapping regions could be linked to the finding of increased functional connectivity in default mode subnetworks.^49,50^ In the context of Alzheimer’s disease, there is a hypothesis suggesting allostatic overload, marked by chronic activation of stress pathways.^131,132^ This chronic activation leads to insulin resistance, associated with Alzheimer’s disease neuropathology, brain function and cognition (particularly, with cognitive aspects linked to the default mode and ventral attention networks, such as memory consolidation^133,134^). Insulin receptors, involved in these processes, are present in multiple brain regions including the hippocampus, amygdala and insula.^131,135^

Our findings indicate functional connectivity alterations in Alzheimer’s disease primarily in the medial temporal lobe, diverging from some previous studies that also report changes in cortical areas like the precuneus and ventromedial prefrontal cortex, and in early-affected regions like the thalamus and locus coeruleus.^34-36,48,54,136^ The absence of identified alterations in subcortical default mode network structures other than the amygdala could be due to a lack of shared data; small sample sizes of some studies; heterogeneous patient groups potentially due to, for example, age differences among studies^137^; or other methodological issues. To gain a clearer understanding of these issues, we recommend future original research studies involving higher-resolution data and larger sample sizes. Notwithstanding these discrepancies, our functional connectivity map for the medial temporal lobe cluster aligns well with the spatial pattern of the default mode network.

The samples of Alzheimer’s disease patients in the included experiments primarily consisted of individuals that typically exhibit noticeable cognitive decline but still retain a degree of independence in daily activities (CDR scores 0.5–1.3).^138^ These individuals average around 12 years of education, often corresponding to high school completion. Depending on the country of origin, this level of education might contribute to cognitive reserve, potentially enhancing compensatory mechanisms reflected in the increased connectivity of the insula.^139^ Such compensatory mechanisms could serve to maintain cognitive function amidst aging and disease onset, as suggested by the Compensation-Related Utilization of Neural Circuits Hypothesis (CRUNCH) and Scaffolding Theory of Aging and Cognition (STAC) models.^140,141^ Alternatively, the increased connectivity of the insula may rather directly stem from the initial pathology.^142,143^ It is also noteworthy that we included studies that did not report Alzheimer’s disease subtypes (e.g. hippocampal sparing). This suggests a more homogenous disease profile.

A potential limitation of this study is that the data are constrained by the availability of coordinates or brain maps in previous research, potentially biasing results towards more commonly reported regions. Coordinates are more often available than whole-brain statistical maps which lead to lower spatial resolution in the data and meta-analysis results, as well as to variability in criteria used to extract coordinates (e.g. amount of peak coordinates per cluster and centre of mass). This makes it difficult to, for example, discern between nuclei of subcortical regions. To address this issue, it is important to promote open research, including the use of open repositories for neuroimaging data such as NeuroVault, openfMRI and OpenNeuro.^62,144,145^ Another limitation is the presence of relatively small sample sizes of about 20 subjects in some studies.^20^ Future studies should also design appropriate ways in which to take into account the differential power of brain regions in functional connectivity.^146^ Methodological limitations in previous studies might also explain why certain subcortical regions are missing from our findings and existing literature. Despite exclusively including studies of all brain regions, with volume- or combined volume- and surface-based analysis approaches, factors like those outlined in our introduction could lead to an underrepresentation of the subcortical the default mode network.^17^ This includes challenges like extracting functional connectivity using signals with low signal-to-noise ratios in subcortical structures and difficulties in aligning small, variable subcortical regions for group analysis.^20,24,59,60^

This study has advanced our understanding of the subcortical structures that make up the default mode network and its alteration in Alzheimer’s disease. This study has also identified both cortical and subcortical brain regions that exhibit functional connectivity changes in Alzheimer’s disease, as well as the direction of the changes and their connection to whole-brain intrinsic functional connectivity. The participation of small subcortical regions and subparts of subcortical regions, as well as the extent of alterations in the subcortical default mode network in Alzheimer’s disease, remains unclear. Further research is needed, for example, expanding our seed-based connectivity analysis to Alzheimer’s disease patients and age-matched controls. It is also important to continue promoting open research practices in order to improve the coverage and unbiased approach of meta-analytical studies.

## Supplementary Material

fcae128_Supplementary_Data

## Data Availability

All brain maps derived from this study are publicly available at https://github.com/Sleosoz/subcortical-DMN-and-Alzheimer.
