# Supplementary material for: The subcortical default mode network and Alzheimer’s disease: a systematic review and meta-analysis
Source: Brain Commun. 2024 Apr 10;6(2):fcae128. doi: 10.1093/braincomms/fcae128 (PMC11043657; doi:10.1093/braincomms/fcae128)
Supplement: fcae128_Supplementary_Data [file fcae128_supplementary_data.zip › supplementary_materials.pdf]

Supplementary material

1 Supplementary methods

1.1 Data Extraction

Coordinates from five of the experiments (corresponding to four studies [1, 14, 10, 2]) were extracted from atlases, and one thresholded t-statistical map provided in the NeuroVault database [6], Cole's lab Github repository, the Choi's striatal parcellation in the Freesurfer wiki, and the Smith's networks at the FMRIB (Oxford) webpage. The coordinates from the five maps obtained from databases were extracted using FMRIB Software Library (FSL) [9], and AFNI tools [3, 4]. The foci of local maxima were extracted in the case of having a t-statistical map [12], and the foci of local centers of mass were extracted taking into account the distance to the cluster boundaries in the case of having an atlas. Coordinates from clusters with sizes greater than 10 voxels were included in the analysis. Coordinates originally reported in Talairach space were transformed into MNI space using the icbm2tal tool implemented by the BrainMap project in their application GingerALE [11]. The GingerALE implementation of this tool transforms coordinates in MNI space to Talairach space, and vice versa.

1.2 Data set used for empirical validation and network analysis

The initial data set consisted of 184 participants, but 12 were excluded due to Quality Control issues identified within the Human Connectome Project (Quality Control issues recognized as A, B, C, and D).

Neuroimaging data were acquired with a Siemens Magnetom 7T MR Scanner and a Nova 32 32-channel Siemens receive head coil from Nova Medical. Two 16-min-long resting-state sessions were used per participant, in which one resting-state session was acquired in an anterior-to-posterior phase direction, and the other resting-state session was acquired in a posterior-to-anterior phase direction (rs-fMRI 1 and 2). Participants were instructed to fix their sight on a white cross-hair over a dark background [15]. The volumes were acquired using Gradient-Echo EPI, with a multiband factor of 5, and 85 slices per volume. The slice thickness was 1.6 mm with no gap, and the field of view (FOV) was 208 x 208 mm. The repetition time (TR) was 1000 ms, echo time (TE) was 22.2 ms, and flip angle was 45 degrees.

1.3 Preprocessing of the data sets used for validation and network analysis

The downloaded HCP 7T data was already preprocessed with the HCP minimal preprocessing pipelines [5]. This included estimating transformations to reduce head motion using FSL MCFLIRT [8], applying fieldmap and gradient distortion corrections, and estimating non-linear transformations from fMRI to MNI space. The pipelines also minimize smoothing by preserving the native space resolution in the transformation to MNI, and by combining and applying all transformations in a single step using sinc interpolation. The data in MNI space were temporally filtered using a 2000 s high-pass filter, and denoised using FIX [7, 13]. The voxels in the resulting data had an isotropic resolution of 1.6 mm. CSF and WM signals were extracted and regressed using each session's `wmparc` file.

2 Supplementary figures

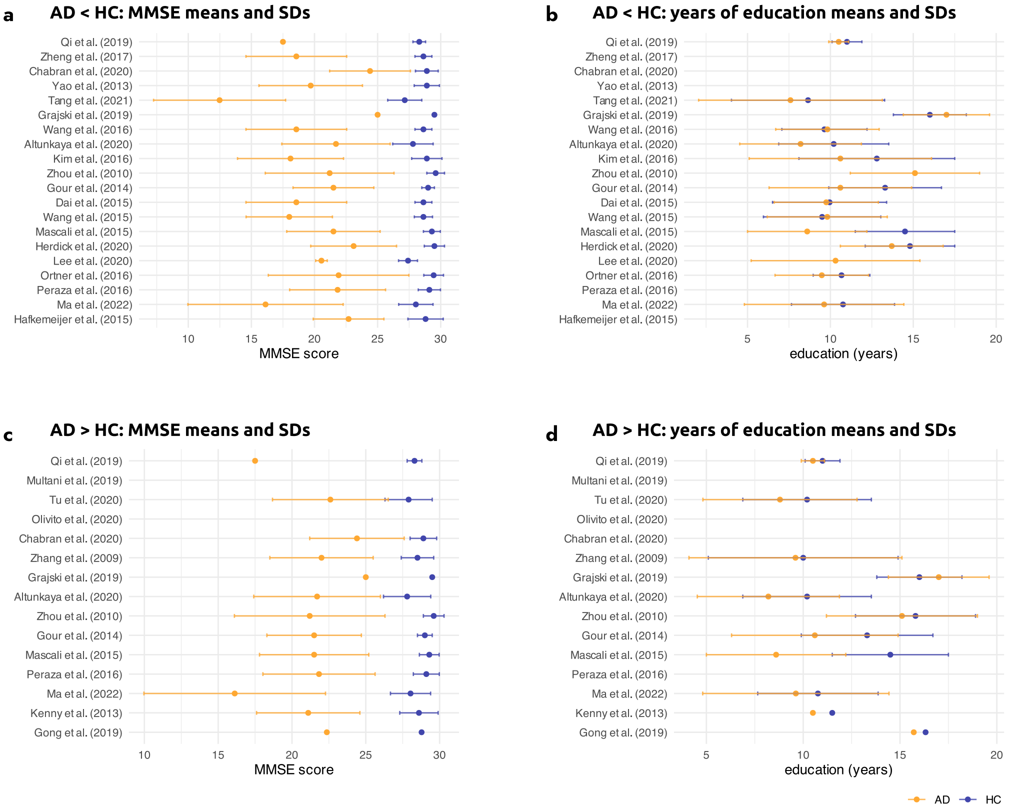

Supplementary Figure 1: Middle values and standard deviations (SDs) of Mini-Mental State Examination scores (MMSE) and years of education, across all studies included in our Alzheimer's disease (AD) meta-analyses. In panels a and b respectively, there are two forest plot representations of middle values and standard deviations for MMSE scores and years of education in the AD < healthy controls (HC) contrast. In panels c and d, there are two forest plot representations of middle values and standard deviations for MMSE scores and years of education in the AD > HC contrast.

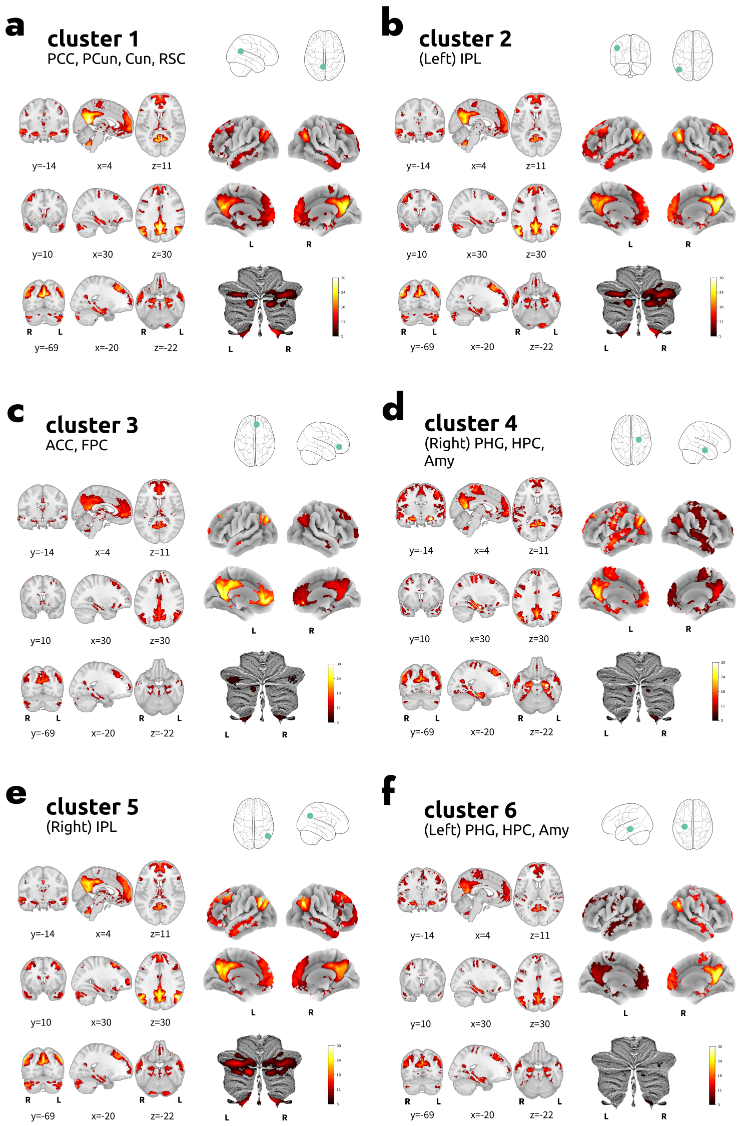

Supplementary Figure 2: Functional connectivity maps from each default mode network region of interest (ROI). From a to f, functional connectivity maps (t-values) of clusters 1 to 6 (voxel-wise one-sample t-tests with 5000 permutations and threshold-free cluster enhancement at p ¡ 0.001).

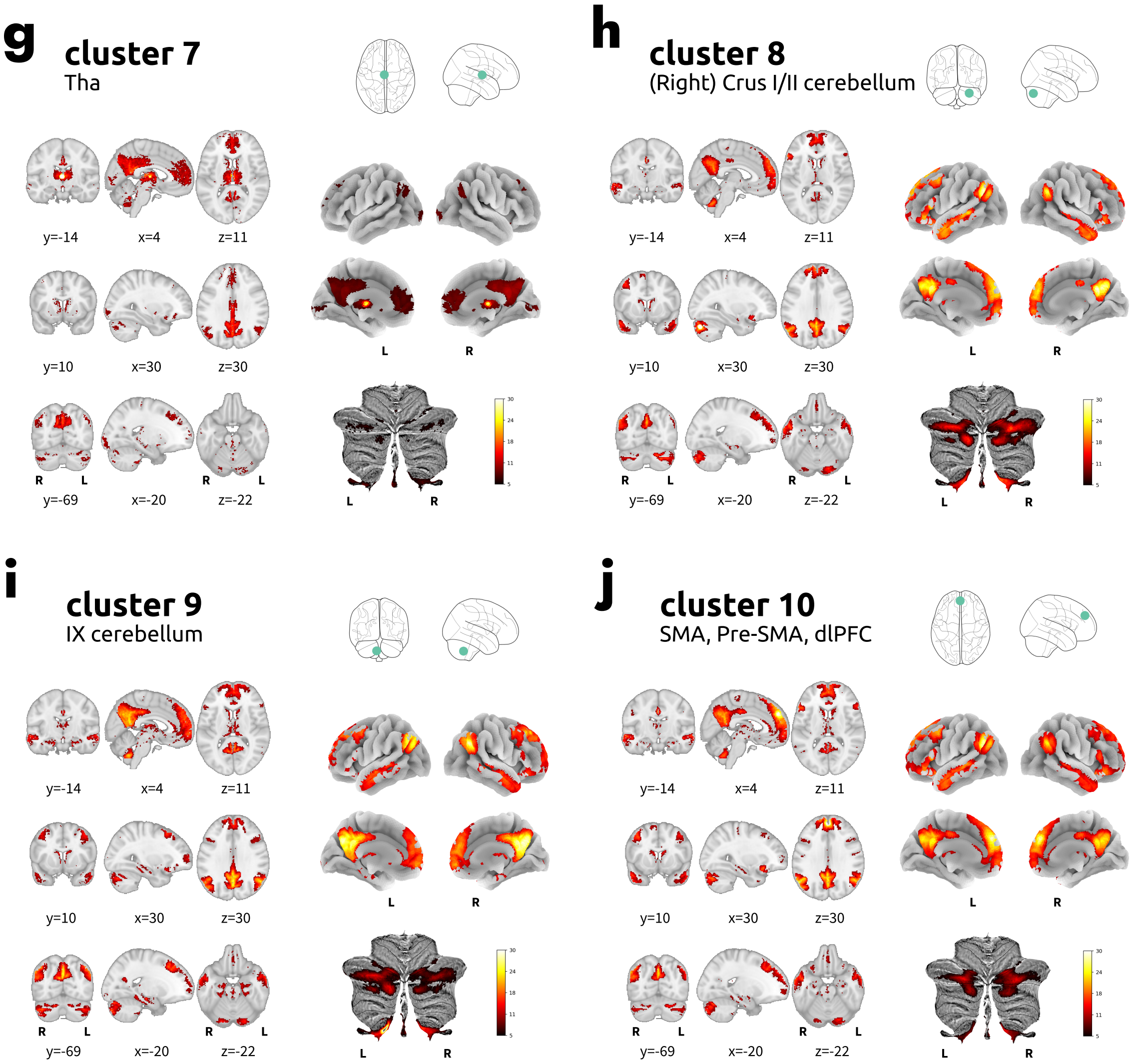

Supplementary Figure 3: Continuation of functional connectivity maps from each default mode network ROI. From g to j, functional connectivity maps (t values) of clusters 7 to 10 (voxel-wise one-sample t-tests with 5000 permutations and threshold-free cluster enhancement at p ¡ 0.001).

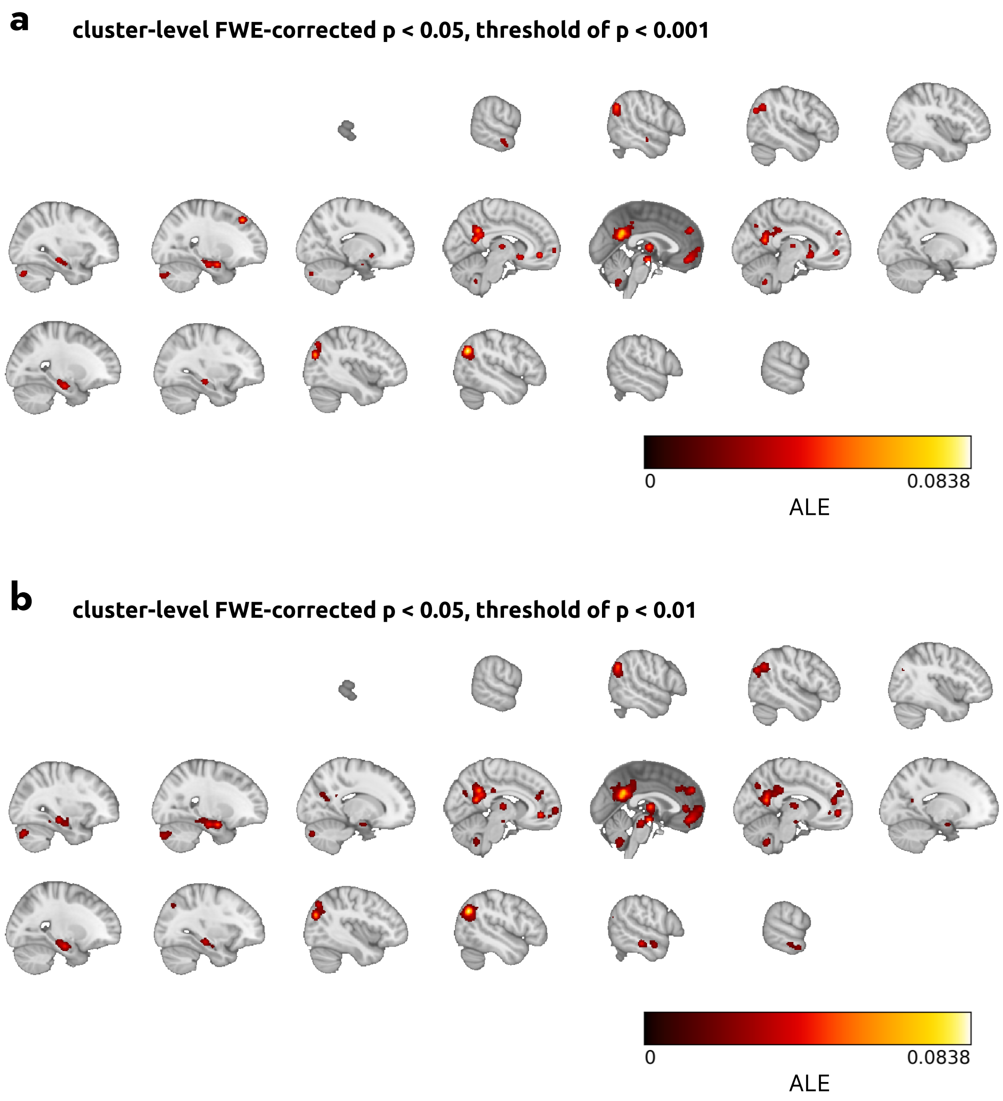

Supplementary Figure 4: Results from the meta-analysis of default mode network brain sites in healthy adults using less stringent thresholds that those reported in our manuscript, presented in radiological convention. In a, cluster-level family-wise error (FWE)-correction $p < 0.05$, with a cluster-forming threshold of $p < 0.001$. In b, cluster-level FWE-correction $p < 0.05$, with a cluster-forming threshold of $p < 0.01$.

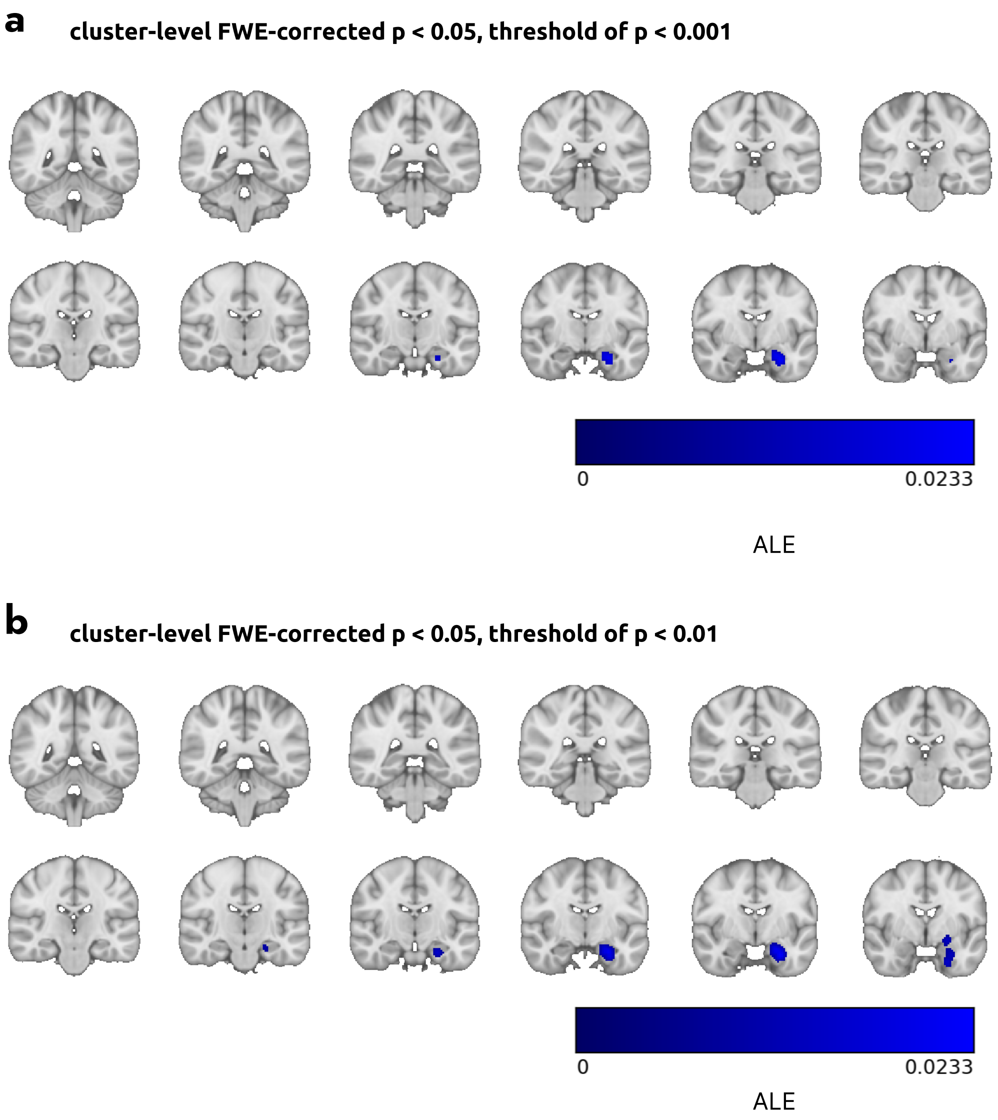

Supplementary Figure 5: Results from the meta-analysis decreases in functional connectivity in Alzheimr's disease patients compared to healthy controls, using less stringent thresholds that those reported in our manuscript, presented in radiological convention. In a, cluster-level FWE-correction $p < 0.05$, with a cluster-forming threshold of $p < 0.001$. In b, cluster-level FWE-correction $p < 0.05$, with a cluster-forming threshold of $p < 0.01$.

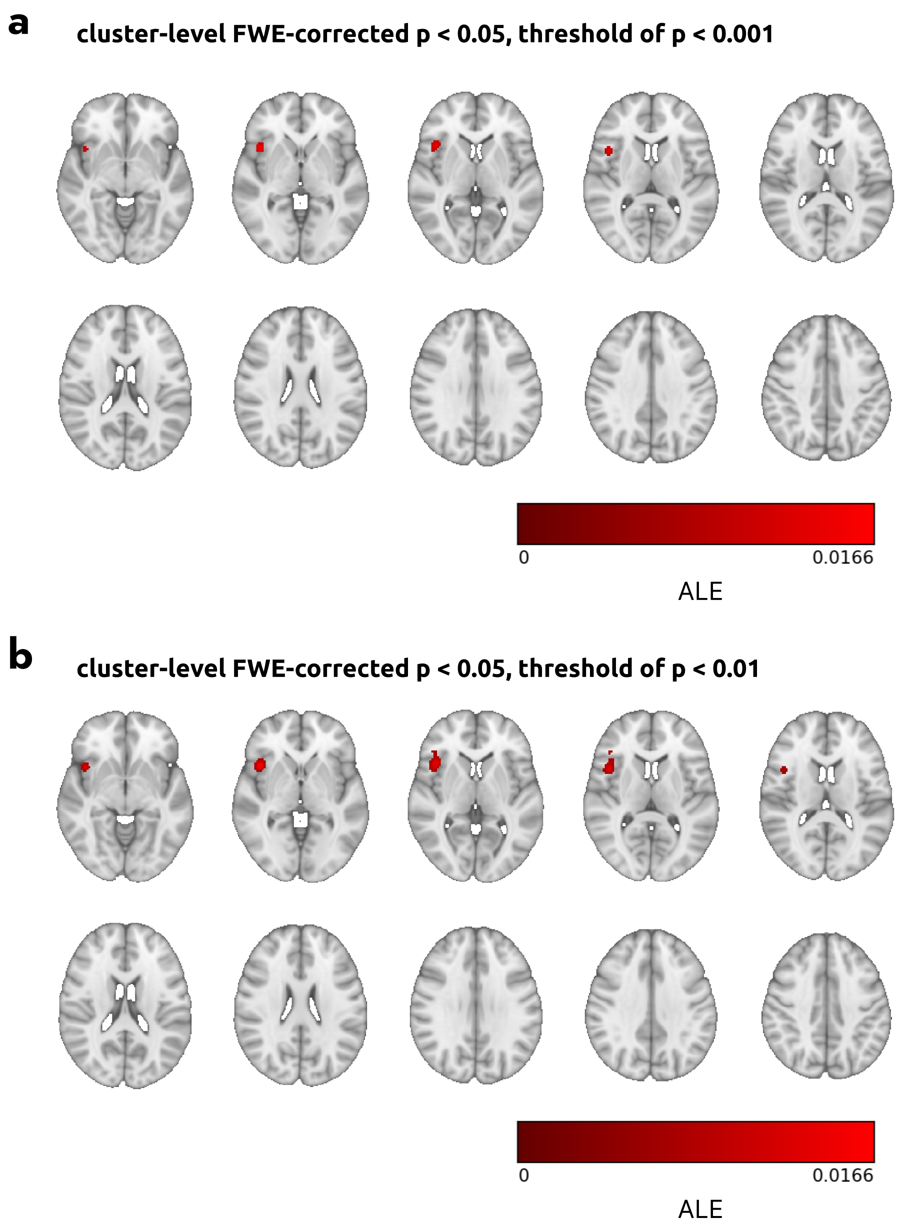

Supplementary Figure 6: Results from the meta-analysis increases in functional connectivity in Alzheimer's disease patients compared to healthy controls, using less stringent thresholds that those reported in our manuscript, presented in radiological convention. In a, cluster-level FWE-correction $p < 0.05$, with a cluster-forming threshold of $p < 0.001$. In b, cluster-level FWE-correction $p < 0.05$, with a cluster-forming threshold of $p < 0.01$.

References

[1] P. N. Alves, C. Foulon, V. Karolis, D. Bzdok, D. S. Margulies, E. Volle, and M. Thiebaut de Schotten. An improved neuroanatomical model of the default-mode network reconciles previous neuroimaging and neuropathological findings. *Commun Biol*, 2:370, Oct. 2019.

[2] E. Y. Choi, B. T. T. Yeo, and R. L. Buckner. The organization of the human striatum estimated by intrinsic functional connectivity. *J. Neurophysiol.*, 108(8):2242–2263, Oct. 2012.

[3] R. W. Cox. AFNI: software for analysis and visualization of functional magnetic resonance neuroimages. *Comput. Biomed. Res.*, 29(3):162–173, June 1996.

[4] R. W. Cox and J. S. Hyde. Software tools for analysis and visualization of fMRI data. *NMR Biomed.*, 10(4-5):171–178, 1997.

[5] M. F. Glasser, S. N. Sotiropoulos, J. A. Wilson, T. S. Coalson, B. Fischl, J. L. Andersson, J. Xu, S. Jbabdi, M. Webster, J. R. Polimeni, D. C. Van Essen, M. Jenkinson, and WU-Minn HCP Consortium. The minimal preprocessing pipelines for the human connectome project. *Neuroimage*, 80:105–124, Oct. 2013.

[6] K. J. Gorgolewski, G. Varoquaux, G. Rivera, Y. Schwarz, S. S. Ghosh, C. Maumet, V. V. Sochat, T. E. Nichols, R. A. Poldrack, J.-B. Poline, T. Yarkoni, and D. S. Margulies. NeuroVault.org: a web-based repository for collecting and sharing unthresholded statistical maps of the human brain. *Front. Neuroinform.*, 9:8, Apr. 2015.

[7] L. Griffanti, G. Salimi-Khorshidi, C. F. Beckmann, E. J. Auerbach, G. Douaud, C. E. Sexton, E. Zsoldos, K. P. Ebmeier, N. Filippini, C. E. Mackay, S. Moeller, J. Xu, E. Yacoub, G. Baselli, K. Ugurbil, K. L. Miller, and S. M. Smith. ICA-based artefact removal and accelerated fMRI acquisition for improved resting state network imaging. *Neuroimage*, 95:232–247, July 2014.

[8] M. Jenkinson, P. Bannister, M. Brady, and S. Smith. Improved optimization for the robust and accurate linear registration and motion correction of brain images. *Neuroimage*, 17(2):825–841, Oct. 2002.

[9] M. Jenkinson, C. F. Beckmann, T. E. J. Behrens, M. W. Woolrich, and S. M. Smith. FSL. *Neuroimage*, 62(2):782–790, Aug. 2012.

[10] J. L. Ji, M. Spronk, K. Kulkarni, G. Repovš, A. Anticevic, and M. W. Cole. Mapping the human brain's cortical-subcortical functional network organization. *Neuroimage*, 185:35–57, Jan. 2019.

[11] J. L. Lancaster, D. Tordesillas-Gutiérrez, M. Martinez, F. Salinas, A. Evans, K. Zilles, J. C. Mazziotta, and P. T. Fox. Bias between MNI and talairach coordinates analyzed using the ICBM-152 brain template. *Hum. Brain Mapp.*, 28(11):1194–1205, Nov. 2007.

[12] C. Maumet and T. Nichols. Generating and reporting peak and cluster tables for voxel-wise inference in FSL. *Res. Ideas Outcomes*, 3:e12368, Feb. 2017.

[13] G. Salimi-Khorshidi, G. Douaud, C. F. Beckmann, M. F. Glasser, L. Griffanti, and S. M. Smith. Automatic denoising of functional MRI data: combining independent component analysis and hierarchical fusion of classifiers. *Neuroimage*, 90:449–468, Apr. 2014.

[14] S. M. Smith, P. T. Fox, K. L. Miller, D. C. Glahn, P. M. Fox, C. E. Mackay, N. Filippini, K. E. Watkins, R. Toro, A. R. Laird, and C. F. Beckmann. Correspondence of the brain's functional architecture during activation and rest. *Proc. Natl. Acad. Sci. U. S. A.*, 106(31):13040–13045, Aug. 2009.

[15] S. M. Smith, C. F. Beckmann, J. Andersson, E. J. Auerbach, J. Bijsterbosch, G. Douaud, E. Duff, D. A. Feinberg, L. Griffanti, M. P. Harms, M. Kelly, T. Laumann, K. L. Miller, S. Moeller, S. Petersen, J. Power, G. Salimi-Khorshidi, A. Z. Snyder, A. T. Vu, M. W. Woolrich, J. Xu, E. Yacoub, K. Uğurbil, D. C. Van Essen, M. F. Glasser, and WU-Minn HCP Consortium. Resting-state fMRI in the human connectome project. *Neuroimage*, 80:144–168, Oct. 2013.
